# Supplementary material for: Vitamin B6, B12, and Folate’s Influence on Neural Networks in the UK Biobank Cohort
Source: Nutrients. 2024 Jun 27;16(13):2050. doi: 10.3390/nu16132050 (PMC11243472; doi:10.3390/nu16132050)
Supplement: Supplementary file 1 [file nutrients-16-02050-s001.zip › nutrients-3056878-supplementary.pdf]

Supplementary Materials

Supplementary Table S1. Interpretation of Independent Components that Constitute Neural Networks in UK Biobank

**Independent Component**	**Neural Network Description**
IC 2	Dorsal and Ventral Stream Visual Network
IC 4	Visuocerebellar Network
IC 8	Primary Visual Network
IC 19	Cerebello-Thalamo-Cortical Network
IC 20	Posterior Default Mode Network

IC2: This network primarily comprises regions associated with the dorsal and ventral visual processing streams. While it does not directly involve the primary visual cortex, the connectivity patterns indicate inputs originating from visual areas and projecting to parietal regions (part of the dorsal stream) as well as inferior temporal areas (part of the ventral stream). **IC4**: The overwhelming activation in this network is localized to the occipital lobe, clearly implicating visual processing functions. Additionally, the network shows robust engagement of the midline cerebellar regions, specifically the archiocerebellum, which is the most evolutionarily conserved part of the cerebellum and is involved in visual-motor integration and balance control. **IC8**: This network is predominantly focused on the primary visual cortex in the posterior regions. However, it also extends into extrastriate visual areas without crossing the boundaries of the occipital lobe. **IC19**: Similar to IC4, this network also exhibits activation in the primary visual cortex and the archiocerebellum. The distinguishing feature is the bilateral involvement of regions in the posterior thalamus, suggesting a cerebellar-thalamo-cortical pathway engagement. **IC20**: Associated with the introspective and mnemonic components of the Default Mode Network, particularly involving the visual-spatial aspects of memory and daydreaming. The activation of this network typically occurs during restful wakefulness and self-generated thought, and may interplay with visual processing regions when visualizing memories or planning.

Supplementary Table S2. Main effects for Folate and Vitamin B12 intake (log transformed)

Neural Network	Folate_log			VB12_log			VB6_log		
	beta	SE	p	beta	SE	p	beta	SE	p
2	0.029966	0.008987	0.000862	-0.03007	0.011376	0.008233	-0.03416	0.019507	0.07997
4	0.031787	0.008555	0.000205	-0.03334	0.010781	0.001993	-0.04354	0.018507	0.018675
8	0.049665	0.013181	0.000167	-0.05028	0.016577	0.002432	-0.0804	0.028189	0.004361
19	0.04326	0.010483	3.75E-05	-0.04878	0.013098	0.000198	-0.08366	0.022327	0.000181
20	0.030733	0.005795	1.19E-07	-0.02387	0.007401	0.001265	-0.04604	0.012648	0.000275

Supplementary Table S3. Main effects for Folate and Vitamin B12 (boxcox-transformed)

Neural Network	Folate_coxbox			VB12_coxbox			VB6_coxbox		
	beta	SE	p	beta	SE	p	beta	SE	p
2	0.000348	0.000162	0.031391	-0.03539	0.013842	0.0106	-0.03763	0.022947	0.101121
4	0.000412	0.000154	0.007403	-0.04016	0.013116	0.002209	-0.04895	0.021765	0.024564
8	0.000643	0.000237	0.006671	-0.0599	0.020167	0.00299	-0.08968	0.033154	0.006853
19	0.000558	0.000188	0.003062	-0.05745	0.015936	0.000315	-0.09472	0.026263	0.000313
20	0.000426	0.000104	4.58E-05	-0.02742	0.009008	0.00235	-0.05014	0.014887	0.000764

Supplementary Table S4. Main effects for Folate and Vitamin B12

Neural Network	Folate_mean			VB12_mean			VB6_mean		
	beta	SE	p	beta	SE	p	beta	SE	p
2	0.000124	6.49E-05	0.05701	-0.00207	0.000733	0.004837	-0.01154	0.004824	0.016817
4	0.000152	6.17E-05	0.014052	-0.00179	0.000695	0.009947	-0.01258	0.004578	0.006026
8	0.000235	9.50E-05	0.013422	-0.00287	0.00107	0.007347	-0.02306	0.006972	0.000949
19	0.000204	7.55E-05	0.006884	-0.00337	0.000844	6.51E-05	-0.02288	0.005522	3.47E-05
20	0.000156	4.19E-05	0.000204	-0.00168	0.000476	0.000429	-0.01143	0.003127	0.000259

Supplementary Table S5. Estimates for Folate and Vitamin B12 (log transformed) interactions by family history

Component	Folate_log				Vitamin B12			
	Family History Negative		Family History Positive		Family History Negative		Family History Positive	
	Beta	P-value	Beta	P-value	Beta	P-value	Beta	P-value
**IC2**	0.0253	0.0000	0.0318	0.0009	-0.0551	0.0000	0.0116	0.3215
**IC4**	0.0250	0.0000	0.0261	0.0049	-0.0607	0.0000	-0.0006	0.9605
**IC8**	**0.0410**	**0.0000**	**0.0668**	**0.0000**	**-0.1006**	**0.0000**	**0.0265**	**0.1210**
**IC19**	0.0524	0.0000	0.0303	0.0046	**-0.0800**	**0.0000**	**0.0046**	**0.7221**
**IC20**	**0.0211**	**0.0000**	**0.0384**	**0.0000**	-0.0404	0.0000	-0.0116	0.1482

Supplementary Table S6. Estimates for Folate and Vitamin B12 (boxcox transformed) interactions by family history

Component	Folate_boxcox				Vitamin B12_boxcox			
	Family History Negative		Family History Positive		Family History Negative		Family History Positive	
	Beta	P-value	Beta	P-value	Beta	P-value	Beta	P-value
**IC2**	0.000392	0.066658	0.0009	0.0222	-0.0552	0.0006	0.0181	0.5118
**IC4**	0.000346	0.089446	0.0009	0.0150	-0.0565	0.0002	0.0008	0.9751
**IC8**	**0.00057**	**0.069302**	**0.0018**	**0.0008**	**-0.0975**	**0.0000**	**0.0387**	**0.3252**
**IC19**	0.000743	0.003019	0.0006	0.1404	**-0.0828**	**0.0000**	**0.0096**	**0.7488**
**IC20**	**0.000149**	**0.282131**	**0.0009**	**0.0004**	-0.0332	0.0014	-0.0106	0.5609
